# Convallatoxin inhibits proliferation and angiogenesis of glioma cells via regulating JAK/STAT3 pathway

**DOI:** 10.1515/biol-2022-1056

**Published:** 2025-04-28

**Authors:** Zhongfei Hao, Yaming Han, Yunfei Bo, Liwen Cao, Huijie Fang, Yufei Zhang, Qingbin Li

**Affiliations:** Department of Neurosurgery, Second Affiliated Hospital of Harbin Medical University, Harbin Medical University, No. 246, Xuefu Road, Nangang District, Harbin, Heilongjiang, 150086, China

**Keywords:** angiogenesis, cell metastasis, convallatoxin, gliomas, JAK/STAT3 pathway

## Abstract

Gliomas can cause nerve cancer-related death, and surgical removal can be challenging. Convallatoxin functioned as anti-proliferation and anti-angiogenesis in cancer cells. However, convallatoxin’s effect on glioma remains unclear. The aim of this study is to investigate the effect of convallatoxin on the proliferation and angiogenesis of glioma cells, and explore the underlying mechanism. Human glioma cell lines U251MG and A172 were treated with 12.5, 25, and 50 nM convallatoxin. Cell proliferation was investigated using the CCK-8 assay and colony formation assay. Migration and invasion were analyzed with transwell assays. Angiogenesis was evaluated using a tube formation assay. The phosphorylation of Janus kinase (JAK) and signal transducer and activator of transcription 3 (STAT3) was measured using Western blots. A xenotransplantation model of nude mice was used to investigate glioma progression. In U251MG and A172 cells, convallatoxin dose-dependently reduced cell viability and colony formation. Convallatoxin suppressed migration and invasion. Similarly, convallatoxin-treated cells had weakened angiogenesis. Convallatoxin downregulated JAK and STAT3 phosphorylation levels. Convallatoxin also inhibited glioma progression in nude mice xenotransplantation models. By inhibiting the JAK/STAT3 signaling pathway, convallatoxin inhibited proliferation, migration, invasion, and angiogenesis of glioma cells, proving to be a promising therapeutic candidate for gliomas.

## Graphical abstract

**Figure:**
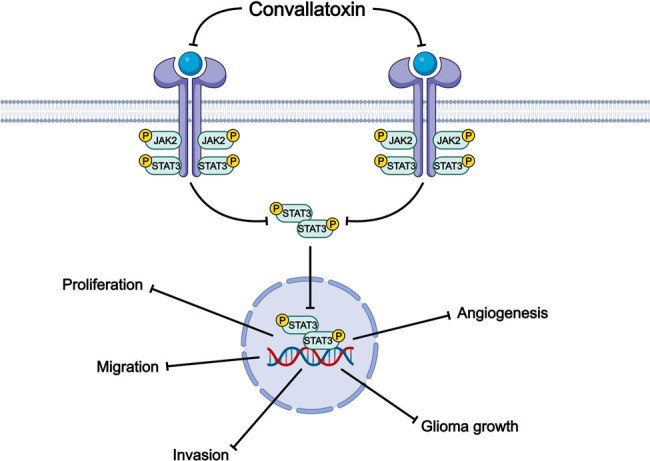

## 1 Introduction

Gliomas account for the majority of tumor-related deaths affecting the central nervous system globally. Angiogenic processes play a role in pathological processes such as tumor development and metastasis [1]. The balance between pro-angiogenic and anti-angiogenic factors plays a crucial role in its regulation. Various diseases, such as cancer, may have an imbalance in pro-angiogenic factors, leading to excessive blood vessel formation and the growth of new blood vessels to sustain the nutritional and oxygen requirements [2]. Both preclinical and clinical studies have validated a variety of angiogenesis inhibitors. There is, however, a risk that these inhibitors may induce tumor adaptation and progression to larger malignancy stages, characterized by heightened invasiveness and metastasis [3]. Therefore, understanding angiogenesis mechanisms facilitates the development of therapeutic strategies that target this process.

Convallatoxin, found in *Convallaria majalis*, is a natural cardiac glycoside [4]. According to *in vitro*, *in vivo*, and early-stage clinical studies [5], convallatoxin exhibits anti-tumor activity. Convallatoxin attenuates the growth, apoptosis, and angiogenesis of colorectal cancer cells [6]. Through down-regulating parathyroid hormone receptor 1 (PTHR1) expression and inactivation of Wnt/β-catenin pathway, convallatoxin inhibits osteosarcoma cell growth, migration, invasion and accelerates osteogenic differentiation [7]. Convallatoxin promotes lung cancer cells’ sensitivity to 5-fluorouracil-mediated cell death by inhibiting apoptosis [8]. These studies illustrate the potential of convallatoxin as a clinical therapy for cancer treatment. Despite its promising potential in *in vitro* cancer treatment, convallatoxin still requires further verification in both *in vitro* and *in vivo* studies, with a long-term perspective on potential clinical applications.

The JAK/STAT3 signaling pathway plays a critical role in cell proliferation, survival, and immune response regulation, making it a significant focus in cancer research. Multiple cancers, including glioma [9], are characterized by abnormal activation of this pathway. In glioma, the JAK/STAT3 pathway contributes to tumor progression and resistance to conventional therapies, highlighting its potential as a therapeutic target [10]. Convallatoxin inhibits STAT3 phosphorylation in colorectal cancer cells, downregulating the expression of angiogenesis-related genes, including vascular endothelial growth factor (VEGF) [6]. Additionally, convallatoxin inhibits the growth of human umbilical vein endothelial cells (HUVEC) and demonstrates anti-angiogenic activity both *in vitro* and *in vivo* [11].

Convallatoxin has not been used in gliomas. In this study, the anti-proliferative and anti-angiogenesis effects of convallatoxin were investigated in human glioma cell lines U251MG and A172, along with its role in migration, invasion, and regulation of the JAK/STAT3 signaling pathway.

## 2 Materials and methods

### 2.1 Cell culture

Two human glioma cell lines U251MG (HTB-17, ATCC, Manassas, Virginia, USA) and A172 (CRL-1620, ATCC) as well as HUVECs (CRL-1730, ATCC) were cultivated in Eagle’s Minimum Essential Medium (ATCC 30-2003) containing 10% fetal bovine serum (FBS) cells were cultured in a 37°C incubator with 5% CO_2_.

### 2.2 Cell viability assay

U251MG and A172 cells were cultured in 96-well plates and treated with 12.5, 25, and 50 nM convallatoxin (C9140, Sigma-Aldrich, St. Louis, MO, USA), respectively. 24 h later, cells were cultivated with new medium supplemented with 10 μL CCK-8 solution (Glpbio, CA, USA) per well and cultured for 24, 48, and 72 h. Cell viability ratio was calculated from absorbance analysis at 450 nm wavelength.

### 2.3 Colony formation assay

U251MG and A172 cells were seeded into six-well plates with 500 cells per well. Based on the cell viability assay, each well was treated with convallatoxin. Once ocular cell clusters were observed, cells were fixed in 4% paraformaldehyde for 15 min and stained with 0.1% violet crystal (548-62-9, Sigma-Aldrich) for 10 min. Colonies were counted after two washings with distilled water, excluding those less than 2 mm in diameter or faintly stained. Total colonies divided by 400 is multiplied by 100 to determine colony-forming efficiency [12].

### 2.4 Transwell assay

U251MG and A172 cells were treated with 12.5, 25, and 50 nM convallatoxin, then seeded in the upper chambers of transwell plates (PI8P01250, Merck, Germany). For the migration assay, no Matrigel (E1270, Sigma-Aldrich) was coated on the inserts; while Matrigel was added to the upper space for the invasion assay. Upper chamber was loaded with serum-free media, while the lower chamber contained 10% FBS and was incubated for 2 days at 37°C. Subsequently, cells that traversed the polycarbonate membrane were fixed using 5% glutaraldehyde and stained with 1% crystal violet. For each sample, over ten randomly selected fields were examined, and average stained cells were quantified.

### 2.5 Tube formation assay

96-well plates were pre-cooled and filled with 50 μL Matrigel Matrix (Corning, Glendale, AZ, USA) and incubated at 37°C for 2 h. HUVECs were cultured with medium from U251MG or A172 cells treated with convallatoxin at densities of 12.5, 25, and 50 nM. After incubating for 24 h, HUVECs were detached using trypsin solution and seeded at 1.5 × 10^4^ cells per well onto the solidified Matrigel layer. Cells were then re-incubated until they adhered and formed tube-like structures on the Matrigel. After 36 h, cells were rinsed with phosphate buffer saline (PBS) and photographed. The branching points of each node were calculated for at least ten randomly selected fields.

### 2.6 Western blot

The total protein of U251 or A172 cells was extracted using lysis buffer (25 mM Tris-HCl pH = 7.4, 250 mM NaCl, 0.5% Sodium deoxycholate, 1 mM EDTA, 0.1% SDS, and 1 mM Phenylmethanesulfonyl fluoride). Lysates were processed for protein-gel running and transferred to nitrocellulose membranes. After blocking with 2.5% bovine serum albumin overnight at 4°C, the membrane was incubated with primary antibodies against p-JAK2 (ab32101, Abcam, Cambridge, United Kingdom, 1:3,000), JAK2 (ab39636, Abcam, 1:800), p-STAT3 (9145, Cell Signaling Technology, Danvers, Massachusetts, USA, 1:2,000), STAT3 (30835, Cell Signaling Technology, 1:1,000), and β-actin (20536-1-AP, ProteinTech, Rosemont, Illinois, USA, 1:5,000) at 4°C overnight. Membrane was then reacted with horseradish peroxidase-conjugated goat anti-mouse IgG (SA00001-1, ProteinTech; 1:5,000) at 37°C for 1.5 h. A ChemiDoc Imaging system (Bio-Rad, Hercules, CA, USA) was used to visualize the membrane using ECL (SolarBio, Beijing, China). Using ImageJ (NIH, USA), the band intensity was normalized to β-actin [13].

### 2.7 Xenotransplantation model of nude mice

A 12-h light/dark cycle and specific pathogen free environment were provided to the BALB/c nude mice purchased from Shanghai Laboratory Animal Center (Shanghai, China). A total of 18 mice were then divided into 3 groups, each consisting of 6 mice. A xenotransplantation model was created by injecting 5 × 10^5^ cells into each nude mouse hypodermically. We used one group of mice as a control and administered 50 μg/kg and 100 μg/kg convallatoxin intraperitoneally to the other two groups, respectively. 20 days later, lymphoid tissues were isolated for measuring diameter and weight [14]. Animal experiments were approved by the Ethics Committee of the Second Affiliated Hospital of Harbin Medical University, and conducted according to the Laboratory Animal Care and Use Guidelines.


**Ethical approval:** The research related to animal use has been complied with all the relevant national regulations and institutional policies for the care and use of animals, and has been approved by the Ethics Committee of Second Affiliated Hospital of Harbin Medical University.

### 2.8 Quantification and statistical analysis

Data analysis was performed using GraphPad Prism 8.0 (Dotmatics, Boston, MA, USA). Data derived from three biological replicates were presented as mean values ± standard deviation (SD), with inter-group differences assessed through unpaired *t*-tests. Statistical analyses for the *in vivo* study were performed using a one-way ANOVA with post-hoc Bonferroni adjustments to compare multiple groups. *p*-value of <0.05 was considered statistically significant.

## 3 Results

### 3.1 Convallatoxin inhibits glioma cell proliferation

**Figure 1:**
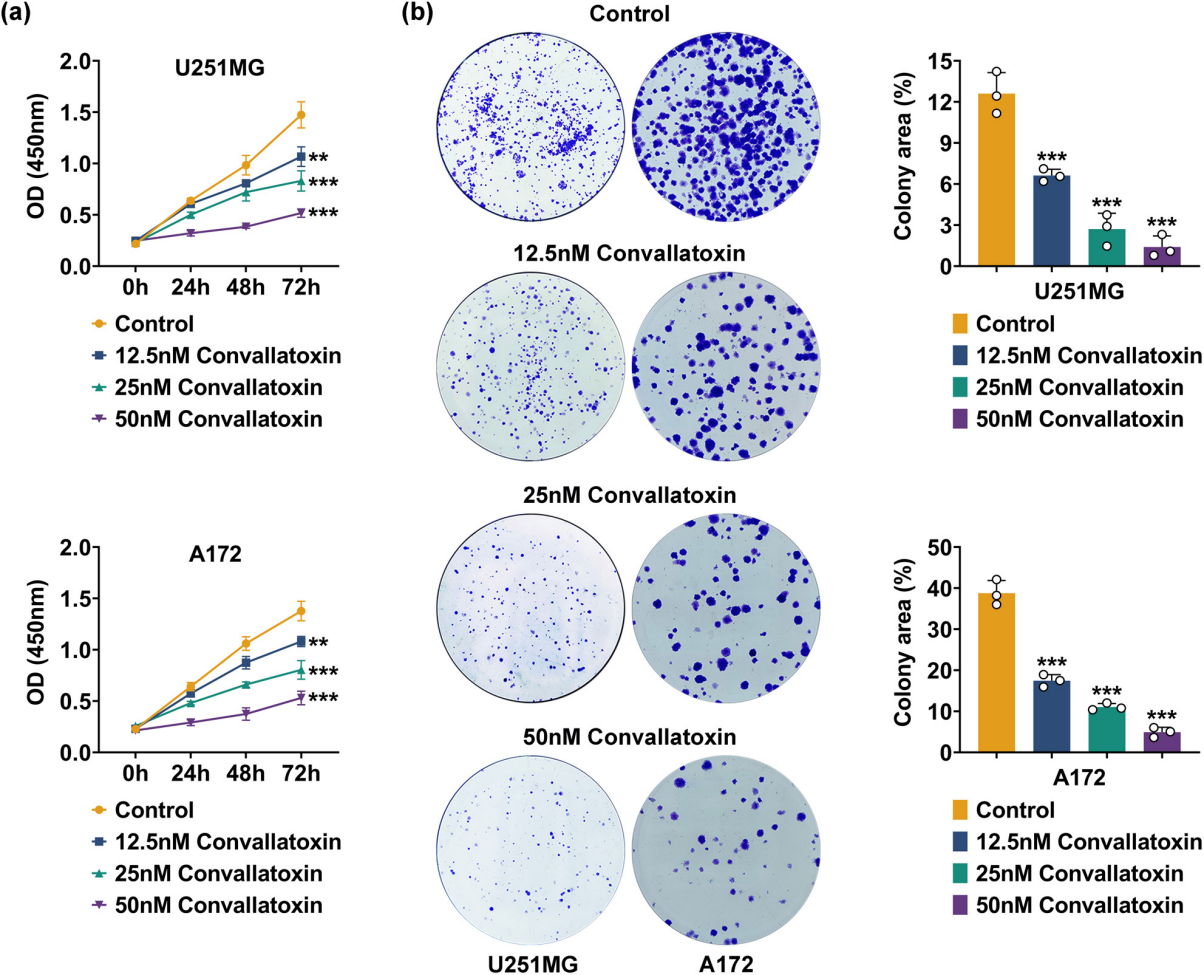
Convallatoxin inhibits glioma cell proliferation. (a) Cell viability of U251MG and A172 treated with convallatoxin for 24, 48, and 72 h. (b) Colony formation in U251MG and A712 cells treated with 12.5, 25, and 50 nM Convallatoxin. Three repeated experiments were analyzed statistically. Error bar, mean value ± SD; * vs Control; ** *p* < 0.01, *** *p* < 0.001.

To uncover the effect of convallatoxin on glioma cells, the cell viability of convallatoxin-treated U251MG and A172 cells was evaluated. Convallatoxin significantly decreased the viability of U251MG and A172 cells at 12.5, 25, and 50 nM (Figure 1a). Convallatoxin also reduced colony formation in U251MG and A712 cells at 12.5, 25, and 50 nM. A negative association was found between the colony counts and convallatoxin concentration (Figure 1b). Therefore, convallatoxin downregulated glioma cell proliferation dose-dependently.

### 3.2 Convallatoxin represses glioma cell invasion and migration

**Figure 2:**
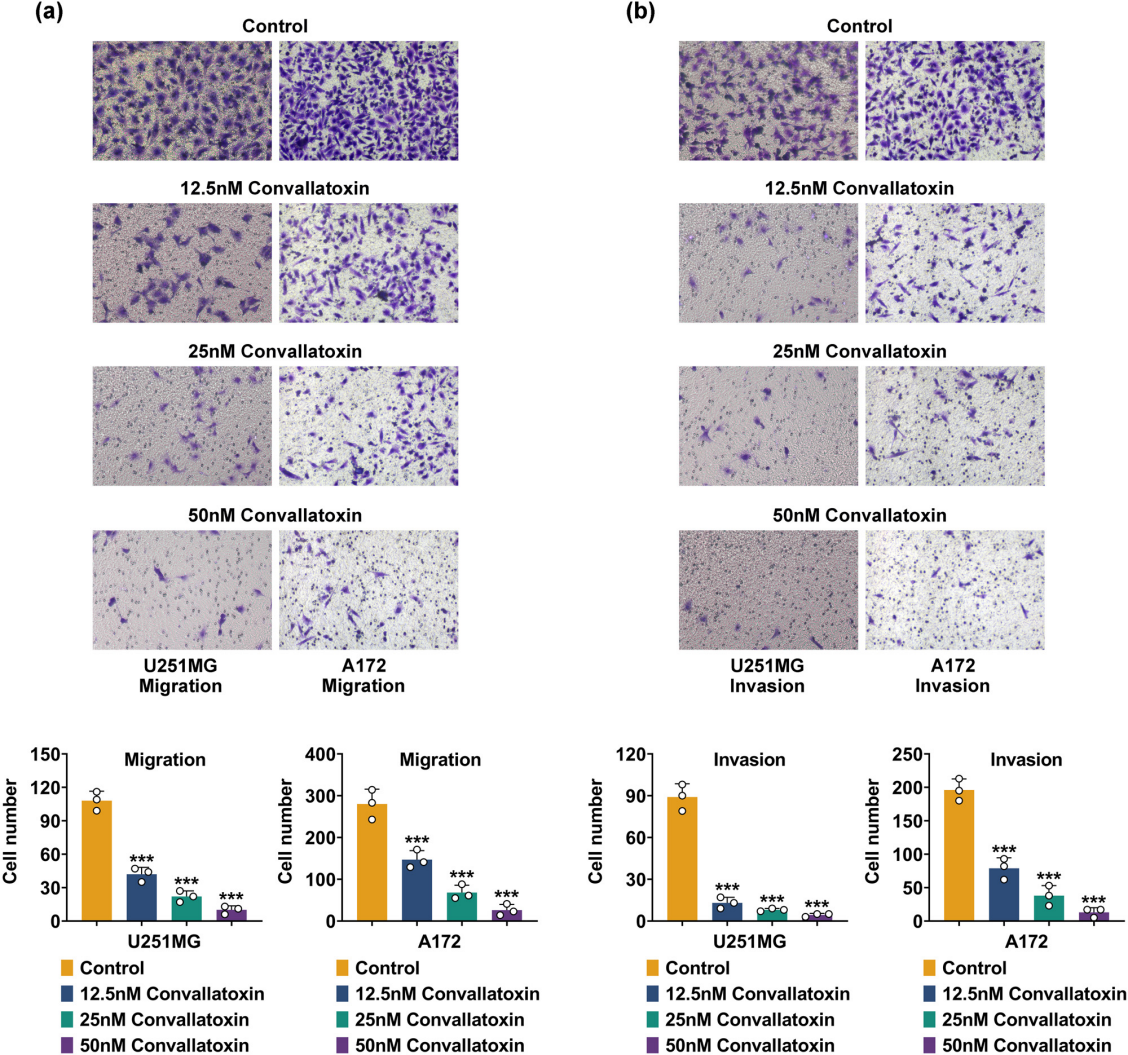
Convallatoxin represses glioma cell invasion and migration. (a) Cell migration analysis of convallatoxin-treated U251 and A172 cells. (b) Cell invasion of U251MG and A172 treated with convallatoxin. Three repeated experiments were analyzed statistically. Error bar, mean value ± SD; * vs Control; *** *p* < 0.001.

Cell migration is crucial for cancer progression, which is necessary during invasion. Both migration and invasion are necessary for tumor metastasis. This study investigated the effect of convallatoxin on migration and invasion. Transwell assays showed that convallatoxin at 12.5, 25, and 25 nM impaired U251 and A172 cell migration (Figure 2a). Convallatoxin significantly reduced the invasion ability of U251MG and A172 cells (Figure 2b). Consequently, convallatoxin inhibited glioma cell invasion and migration.

### 3.3 Convallatoxin weakens glioma cell angiogenesis

**Figure 3:**
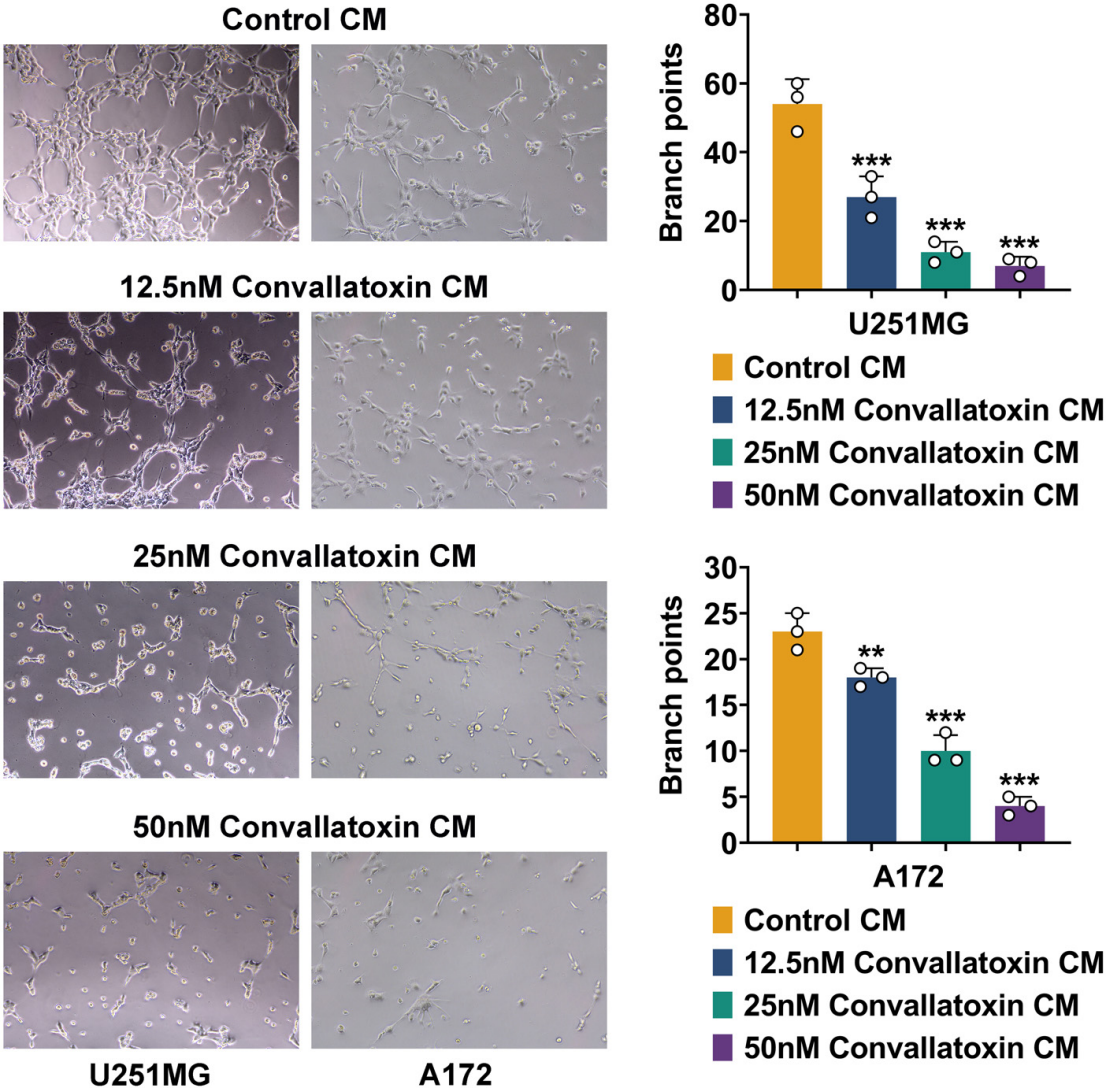
Convallatoxin weakens glioma cell angiogenesis. The tube formation analysis of HUVECs cocultured with culture medium of convallatoxin-treated U251MG and A172 cells. Compared to control CM, the quantity of branch points was negatively related to convallatoxin concentration. Three repeated experiments were analyzed statistically. Error bar, mean value ± SD; * vs Control CM; ** *p* < 0.01, *** *p* < 0.001; CM, culture medium.

Angiogenesis plays a crucial role in glioma tumor growth. For analysis, 12.5, 25, or 50 nM convallatoxin-treated U251MG and A172 cells were cultured with HUVECs, and branch points were counted. Compared to control culture medium (CM), HUVECs cocultured with convallatoxin-treated CM had significantly fewer branch points, and the amount of branch points was negatively correlated with the concentration of convallatoxin (Figure 3), suggesting that convallatoxin suppressed angiogenesis in glioma cells dose-dependently.

### 3.4 Convallatoxin blocks the JAK/STAT3 pathway

**Figure 4:**
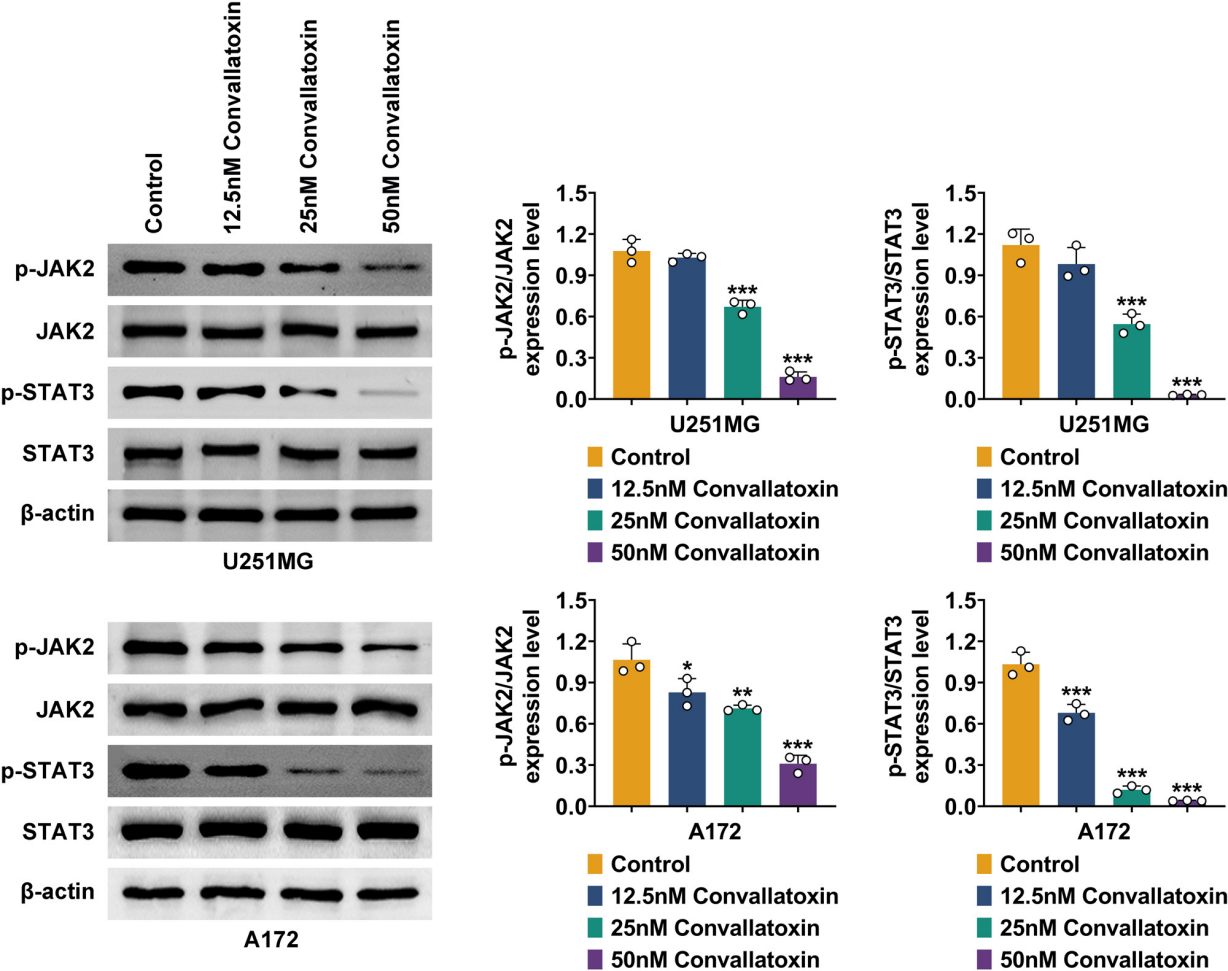
Convallatoxin blocks the JAK/STAT3 pathway. Western blot analysis of JAK and STAT3 protein expression and phosphorylation in convallatoxin-treated U251MG and A172 cells. Three repeated experiments were analyzed statistically. Error bar, mean value ± SD; * vs Control; * *p* < 0.05, ** *p* < 0.01, *** *p* < 0.001.

JAK/STAT3 is a key signaling pathway regulating glioma pathogenesis and progression. In this study, the effect of convallatoxin on the JAK/STAT3 pathway was investigated. Western blots were performed to evaluate JAK and STAT3 protein expression and phosphorylation levels. In U251MG and A172 cells treated with convallatoxin, JAK2 and STAT3 expression was consistent, while their phosphorylation was downregulated. In U251MG cells, 12.5 nM convallatoxin did not reduce the phosphorylation of JAK2 (p-JAK2) or STAT3 (p-STAT3). However, convallatoxin at 25 and 50 nM significantly reduced p-JAK2 and pSTAT3. In A172 cells, convallatoxin at 12.5, 25, and 50 nM significantly decreased p-JAK2 and pSTAT3 (Figure 4). Overall, convallatoxin inhibited the JAK/STAT3 pathway by reducing phosphorylation of JAK and STAT3.

### 3.5 Convallatoxin inhibits gliomas by modulating the JAK/STAT3 signaling pathway

**Figure 5:**
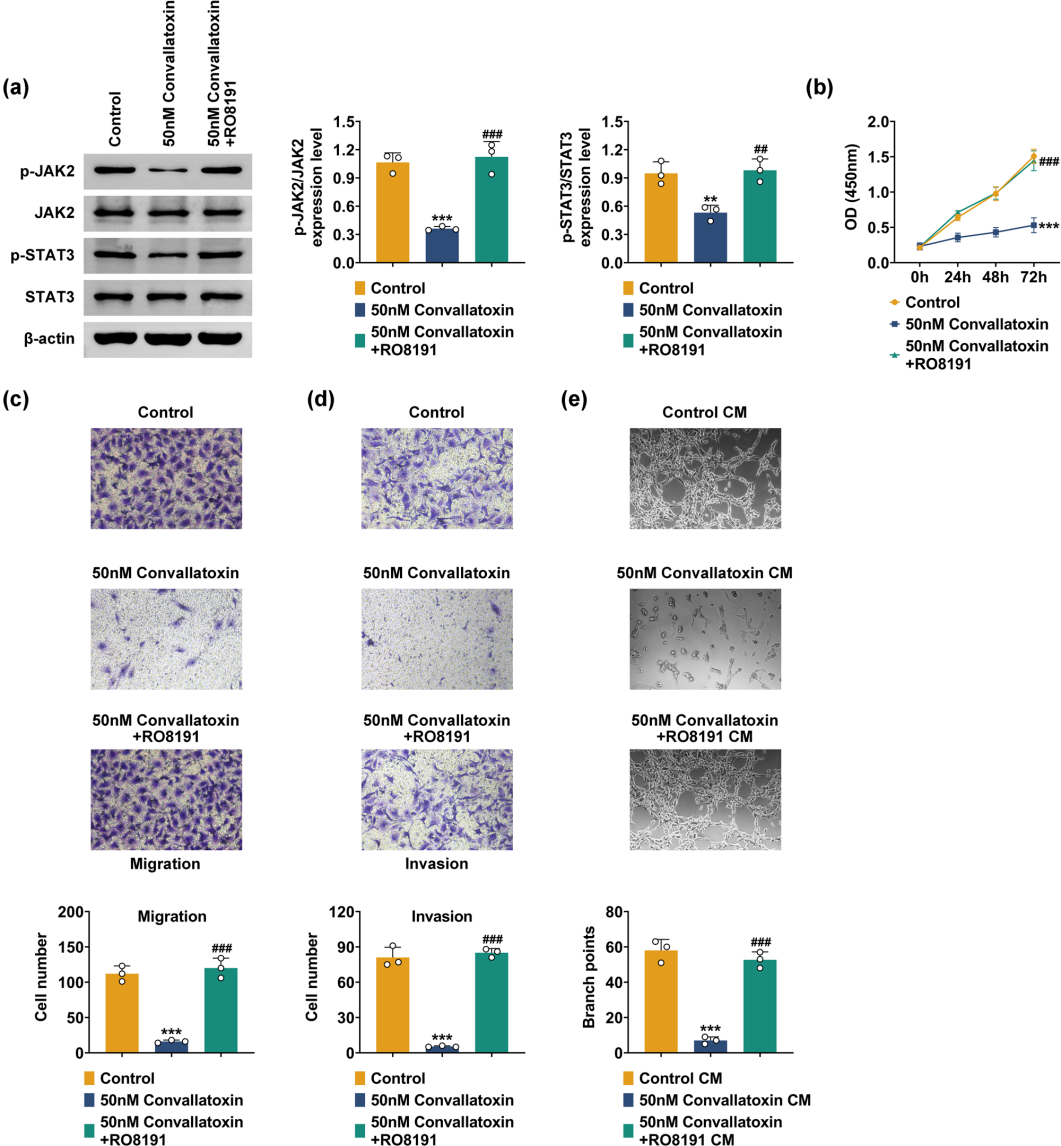
Convallatoxin inhibits gliomas via the JAK/STAT3 signaling pathway. (a) Phosphorylation levels of JAK2 and STAT3 in U251 cells treated with the JAK/STAT3 inhibitor RO8191. (b) RO8191 reversed the convallatoxin-induced decrease in cell proliferation. (c) Migration of U251 cells treated with convallatoxin and RO8191. (d) Invasion of U251 cells treated with convallatoxin and RO8191. (e) Blood vessel branching points induced by convallatoxin were reversed by RO8191. Three repeated experiments were analyzed statistically. Error bar, mean value ± SD; * vs Control; * *p* < 0.05, ** *p* < 0.01, *** *p* < 0.001.

To explore whether convallatoxin inhibits gliomas by modulating the JAK/STAT3 signaling pathway, U251 cells were treated with the JAK/STAT3 inhibitor RO8191. Convallatoxin treatment reduced JAK2 and STAT3 phosphorylation levels significantly, which were restored to near-control levels by RO8191 (Figure 5a). Moreover, RO8191 reversed the convallatoxin-induced decrease in cell proliferation, bringing it back to the control level (Figure 5b). Similarly, RO8191 treatment restored to near-control levels the reduced migration and invasion caused by convallatoxin (Figure 5c and d). Co-culture experiments with tumor cell matrices and HUVECs further demonstrated that the reduction in blood vessel branching points induced by convallatoxin was also reversed to the control level after RO8191 treatment (Figure 5e). Thus, convallatoxin suppresses gliomas by regulating the JAK/STAT3 signaling pathway.

### 3.6 Convallatoxin suppresses glioma growth *in vivo*

**Figure 6:**
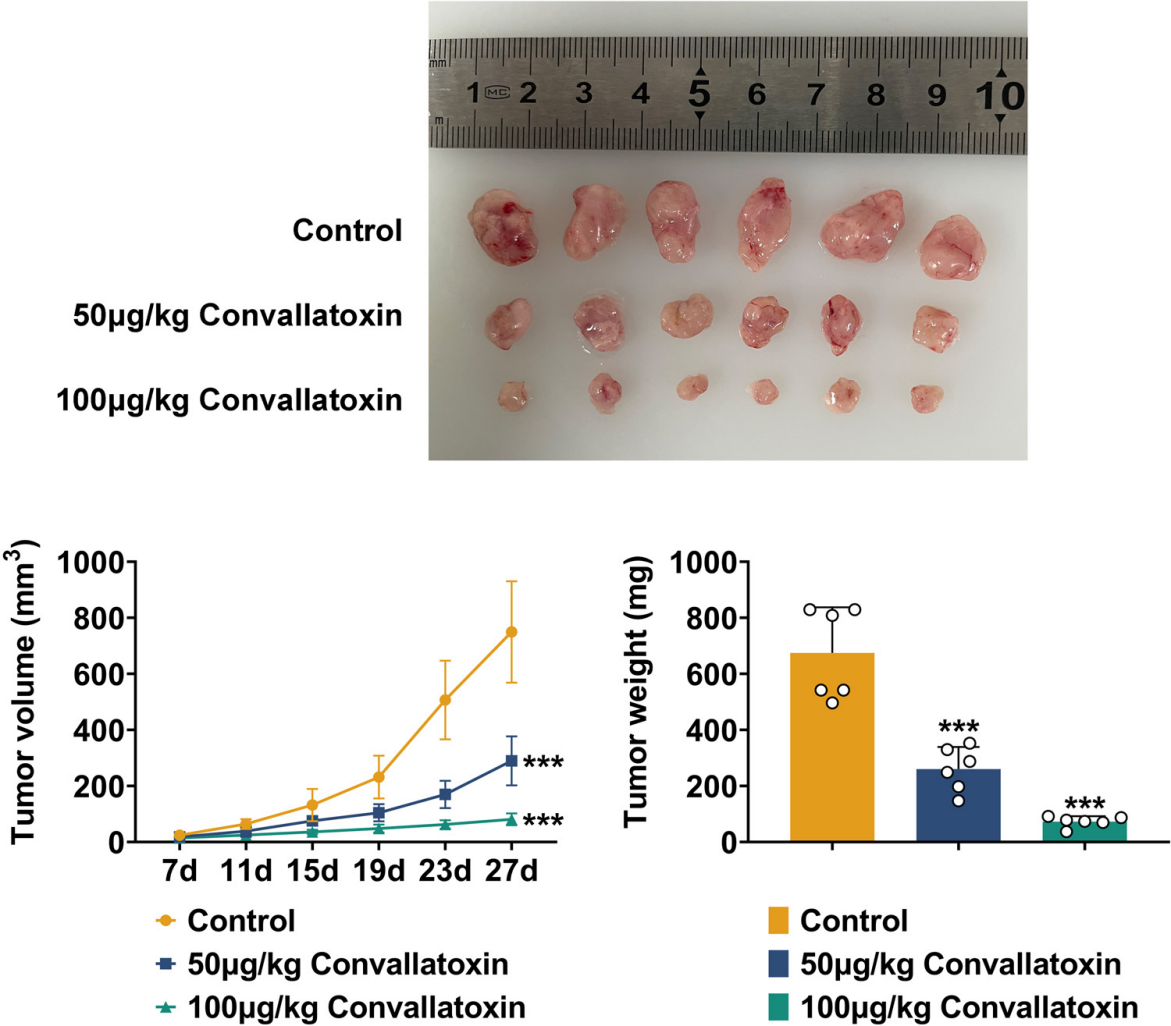
Convallatoxin suppresses glioma growth *in vivo*. The impact of convallatoxin on gliomas was analyzed in the xenotransplantation model in nude mice. 50 and 100 μg/kg convallatoxin significantly reduced tumor tissue diameter and weight. Three repeated experiments were analyzed statistically. Error bar, mean value ± SD; * vs Control; *** *p* < 0.001.

To further study the inhibitory effect of convallatoxin on gliomas, the xenotransplantation model in nude mice was established by injecting convallatoxin-treated glioma cells into nude mice. Compared to the control group, convallatoxin of 50 and 100 μg/kg significantly reduced the diameter of tumor volume. Additionally, mice injected with convallatoxin-treated cells had reduced tumor tissue weight (Figure 6). Thus, convallatoxin inhibits glioma growth *in vivo*.

## 4 Discussion

Gliomas can cause significant damage and impact various functions of the brain due to their invasive nature and ability to disrupt normal brain tissue. It is an aggressive tumor that is difficult to remove completely through surgery. In colorectal cancer, convallatoxin suppresses angiogenesis and growth [6]. By inhibiting apoptosis, convallatoxin advances the death of lung cancer cells induced by 5-fluorouracil [8]. However, convallatoxin’s effects on glioma remain unclear.

In this study, two human gliomas cell lines U251MG and A172 were treated with convallatoxin at doses ranging from 12.5 to 50 nM, and the anti-proliferative and anti-angiogenesis effects were investigated. Cell viability and colony formation assays confirmed the inhibitory role of convallatoxin in glioma cell proliferation.

Convallatoxin exhibits anticancer properties by inhibiting the migration and invasion of cancer cells. Among the mechanisms through which convallatoxin suppresses cancer cell migration and invasion include inhibition of Na^+^/K^+^-ATPase, modulation of epithelial-mesenchymal transition (EMT), and targeting of signaling pathways. Through inhibiting the Na^+^/K^+^-ATPase enzyme, convallatoxin disrupts ion gradients across the cell membrane and alters intracellular calcium levels, leading to changes in cell migration and invasion [15]. EMT facilitates tumor cells to invade surrounding tissues, which is critical for cancer cell invasion and metastasis. Convallatoxin’s inhibition of EMT can prevent the invasive behavior of cancer cells [5]. Furthermore, convallatoxin modulates several signaling pathways involved in cell migration and invasion. For example, convallatoxin inhibits the PI3K/Akt signaling pathway, which is frequently dysregulated in cancers and promotes cell migration and invasion [6,16]. Convallatoxin inhibits osteosarcoma cell growth, migration, invasion, and accelerates osteogenic differentiation by downregulating PTHR1 expression and inactivation of Wnt/β-catenin pathway [7]. By interfering with these pathways, convallatoxin reduces cancer cell migration and invasion. Accordingly, convallatoxin inhibits the migration and invasion of glioma cells.

Angiogenesis feeds and oxygenates the tumor mass, and is therefore crucial for its development and metastasis. Angiogenesis inhibition can effectively limit tumor growth and metastasis [17].

Convallatoxin inhibits angiogenesis in human triple-negative breast cancer by downregulating matrix metalloproteinases and causing cell apoptosis [18]. Convallatoxin inhibits the growth of HUVEC and demonstrated anti-angiogenic activity both *in vitro* and *in vivo* [11]. Angiogenesis and remodeling of the extracellular matrix surrounding blood vessels are important functions of pro-angiogenic factors. By inhibiting their expression, convallatoxin interferes with angiogenic process. Aside from disrupting angiogenesis, convallatoxin can promote apoptosis in these cells.

JAK/STAT3 is a key signaling pathway regulating glioma pathogenesis and progression [19]. By inactivation of the JAK/STAT3 signaling axis, activin receptor-like kinase 4 (ALK4) represses cell growth and migration in glioma [20]. Ribosomal protein L34 (RPL34) knockdown represses glioma cells’ growth and migration via inhibiting the JAK/STAT3 pathway [21]. In colorectal cancer, convallatoxin suppresses proliferation and angiogenesis via cooperation between the mTOR/STAT3 and JAK2/STAT3 pathways [6]. In this study, convallatoxin was found to downregulate the phosphorylation level of JAK and STAT3 when it was applied to the JAK/STAT3 pathway. Phosphorylation of JAK and STAT3 is a critical step in the JAK/STAT signaling pathway, whose dysregulation has been implicated in various diseases, including cancer. To inhibit abnormal cell growth and proliferation, many therapeutic approaches target the JAK/STAT pathway. By inhibiting the JAK/STAT3 pathway, convallatoxin could be a promising treatment for gliomas.

The mechanism by which convallatoxin regulates the JAT/STAT3 signaling pathway remains unclear, and further research is needed. Further, *in vivo* experiments are lacking, particularly regarding convallatoxin’s effects on glioma. The optimal dosage, the route of administration, potential side effects, and drug sensitivity must also be clarified. In summary, convallatoxin attenuates glioma cells’ proliferation, migration, invasion, and angiogenesis via blocking the JAK/STAT3 signaling pathway. Convallatoxin may be a potential treatment for gliomas.
